# Acupuncture Treatment for Cough-Variant Asthma: A Meta-Analysis

**DOI:** 10.1155/2021/6694936

**Published:** 2021-04-02

**Authors:** Jian Xiong, WenChuan Qi, Han Yang, SiTing Zou, Jing Kong, ChengLong Wang, YuanFang Zhou, FanRong Liang

**Affiliations:** ^1^Chengdu University of Traditional Chinese Medicine, Chengdu 6100752, Sichuan, China; ^2^Guangxi University of Traditional Chinese Medicine, Nanning 5300013, Guangxi, China; ^3^The People's Hospital of Guangxi Zhuang Autonomous Region, Nanning, Guangxi 168600, China

## Abstract

**Background:**

In recent years, there have been many clinical reports on acupuncture treatment of cough-variant asthma, but no researcher has objectively analysed and evaluated the efficacy and safety of acupuncture treatment of cough-variant asthma from the perspective of evidence-based medicine.

**Objective:**

To systematically evaluate the clinical efficacy and safety of acupuncture in treating cough-variant asthma and to provide reference values for clinical decision-making.

**Methods:**

The comprehensive computer retrieval Chinese journal full-text database (CNKI), Chinese science and technology periodical database (VIP), ten thousand data knowledge service platform (WanFang Data), PubMed, Embase, and the Cochrane Library were used to collect literature for relevant randomized controlled trials (RCT) of acupuncture treatment of cough-variant asthma, as well as to retrieve papers and add reference retrieval after literature review, in accordance with the standard of literature filtering, data extraction, and quality evaluation. The data were meta-analysed using ReviewManager5.3 software recommended by Cochrane.

**Results:**

A total of 11 randomized controlled clinical studies were screened and included, comprising 929 patients. The results of the meta-analysis showed that, compared with the control group, acupuncture intervention on CVA could enhance the total clinical effectiveness rate, reduce the relapse rate of drug withdrawal, relieve symptoms of cough, phlegm, and diaphragmatic congestion, and improve lung function-related indicators and immune inflammation indicators. There were statistically significant differences in all efficacy evaluation criteria.

**Conclusion:**

The clinical curative effect of acupuncture treatment for cough-variant asthma is precise and has certain advantages in relieving symptoms and reducing the recurrence rate. However, the low quality of the evaluation in the RCT research literature is a problem, and more high-quality clinical randomized controlled trials are needed to further verify the comprehensive clinical efficacy and safety of this treatment. Registration number: PROSPERO (no. CRD42020155244) (https://www.crd.york.ac.uk/prospero/).

## 1. Introduction

Cough-variant asthma (CVA) is a common special type of asthma. Chronic cough is the main or the only manifestation of respiratory disease in the clinic. It is difficult to distinguish and easily misdiagnosed as bronchitis or respiratory infection. The main clinical manifestations of this disease are episodic night cough with no wheezing, and the cough symptoms can be alleviated by hormone therapy [1]. CVA is characterized by hidden onset, a long course, and repeated illness, which directly affects the quality of study, life, work, and sleep of patients and brings great economic and mental burdens to patients and their families. In recent years, due to environmental pollution and the increase in atypical pathogen infection, the incidence of CVA has been on the rise year by year, which has attracted wide attention from the medical community. An investigation on the aetiology of 704 patients with chronic cough in 5 regions of China showed that CVA accounted for the highest proportion, 32.6%, of the aetiology of chronic cough, which is one of the most important causes of chronic cough in China [2].

Current methods of modern medicine in the treatment of CVA are similar to those of asthma, mainly including inhaled bronchodilator, antihistamines, leukotriene receptor antagonist, and glucocorticoid drugs. The method of [3] can achieve effects but has a long period of treatment and easy relapse after drug withdrawal. The ideal treatment has a long-term curative effect, and use of systemic and local application of immune regulators for a long time has side effects similar to corticosteroids; there is resistance and poor treatment adherence on the part of the patient and family for use of glucocorticoid hormones drug treatment [4].

Acupuncture therapy, as an important part of traditional Chinese medicine, has the characteristics of quick effects, low recurrence rates, and few side effects, an especially important value in pain control [5, 6]. In China, this therapy has certain potential and advantages in the direct treatment of CVA and enhancement of curative effects as an auxiliary means. In recent years, there have been many clinical reports on acupuncture treatment of CVA, but no researcher has objectively analysed and evaluated the efficacy and safety of acupuncture treatment of cough-variant asthma from the perspective of evidence-based medicine. Therefore, a meta-analysis was conducted of the literature of randomized controlled trials (RCTs) of acupuncture treatment for cough-variant asthma published in various databases to provide a reference for the formulation and practice of CVA clinical research plans for acupuncture treatment in the future.

## 2. Materials and Methods

### 2.1. Inclusion Criteria for Literature

1. Literature type was published randomized controlled trials (RCT) on acupuncture treatment of cough-variant asthma
2. With clear diagnostic criteria, the subjects were clearly diagnosed as patients with cough-variant asthma
3. In the literature, the intervention measures of the treatment group were acupuncture or acupuncture combined with other therapies, while the control group was placebo or oral nonacupuncture therapy such as Western medicine and Chinese medicine
4. In the same study, when the experimental group was acupuncture combined with other treatment methods, the intervention measures adopted by the control group, except no acupuncture intervention, must be the same as the experimental group
5. Languages were limited to Chinese and English

### 2.2. Literature Exclusion Criteria

1. Studies without definite diagnostic criteria for cough-variant asthma
2. Research on animal experiments
3. Repeatedly published literature
4. Incomplete literature data, or obvious errors
5. Nonclinical randomized controlled trials
6. Theoretical research, case reports, literature reviews, and experience summaries of famous doctors, etc

### 2.3. Literature Retrieval

The Chinese journal full-text database (CNKI), Weipu Chinese Science and Technology journal full-text database (VIP), WanFang data knowledge service platform (WanFang Data), PubMed, Embase, and the Cochrane Library were used to collect and sort out RCT related literature on acupuncture treatment of cough-variant asthma officially published by various data platforms. The retrieval time was designed from the beginning of database construction to 2020-11-23. Chinese search words (“针刺”OR“针刺”OR“电针”OR“耳针”OR“火针”OR“放血”OR“体针”OR“腹针”) AND (“咳嗽变异性哮喘”OR“隐匿性哮喘”OR“咳嗽性哮喘”OR“过敏性咳嗽”OR“咳嗽型哮喘”OR“过敏性支气管炎”) were used (English equivalents: “acupuncture” OR “acupuncture therapy” OR “electroacupuncture” OR “electroacupuncture therapy” OR “manual acupuncture” OR “dry needle” OR “acupoint”) AND (“Cough-variant asthma” OR “Cough Type Asthma” OR “CVA”) Table 1.

### 2.4. Outcome Assessment Indicators

Main outcome measures: total clinical response rate; secondary outcome assessment indicators: (1) recurrence rate; (2) symptom score: cough symptom score, sputum symptom score, and diaphragmatic fullness score; (3) lung function: PEF, FVC, and FEV1; biochemical indicators: CRP, TNF-*α*, and IgE.

### 2.5. Risk Bias and Quality Assessment

The methodological quality assessment of the included studies used the ReviewManager5.3 software risk bias assessment tool provided by the Cochrane collaboration. The evaluation mainly includes eight aspects: the generation of random sequences, the allocation and concealment scheme, the blindness of researchers and subjects, the blind evaluation of study outcome evaluators, the integrity of outcome data, the selective reporting of outcomes, and other sources of bias risk. Two evaluators (Zou and Wang) independently evaluated and cross-checked the performance of the included research design for the above 8 evaluation items and gave the judgement of low risk, unknown risk, and high risk item by item. When there were different evaluation items scores, the two evaluators discussed or the third evaluator (Kong) negotiated to solve the problem. Potential publication bias was analysed by drawing a funnel plot of acupuncture treatment for cough-variant asthma.

The Jadad scoring scale [7] was used to evaluate the included RCTs in three aspects (1–5 points). Low-quality research was 1–2 points, and high-quality research was 3–5 points. The evaluation contents include the following. (1) Random sequence: 2 points were given for the correct description of the random grouping method in the study, and 1 point was given for the “random grouping” and “random” but not describing the method. (2) Blind method: the intervention implementer and the subject in the study describing the blind method correctly was counted as 2 points, and mentioning the “double-blind” method counted as 1 point. (3) Withdrawal and withdrawal: describing the number of withdrawals or loss of follow-up cases and explaining the reasons was worth 1 point.

### 2.6. Statistical Analysis

Meta-analysis, evaluation, and the combined effect of all were performed using Cochrane ReviewManager5.3 software, according to the research into the literature of mainly prospective randomized controlled studies. Count variable data were assessed using the relative risk (RR) and 95% confidence interval for effectiveness analysis of statistics. Continuous variable data were assessed using the MD odds ratio (mean difference) and 95% confidence interval for effectiveness analysis of statistics. The statistically set inspection standard was *P* < 0.05. *P*=0.1 was set as the heterogeneity difference level of the literature, and *P* < 0.1 was considered to reflect the existence of heterogeneity. At the same time, significance index *I*^2^ = 50% was set for quantitative analysis of heterogeneity, where *I*^2^ > 50% indicates that the heterogeneity of all included clinical studies is large. The REM (random-effects model) was used for analysis. Conversely, The FEM (fixed-effects model) was used for analysis. Descriptive analysis was conducted if statistical tests showed significant heterogeneity among the included studies. The objective of this study was to evaluate acupuncture, including the study design of acupuncture as the main or auxiliary treatment for CVA. Therefore, the classification and subgroup analysis was mainly conducted according to the design of the intervention measures to reduce the risk of bias. Sensitivity analysis was applied to test the stability of the results according to the research needs.

## 3. Results

### 3.1. Retrieval

**Figure 1:**
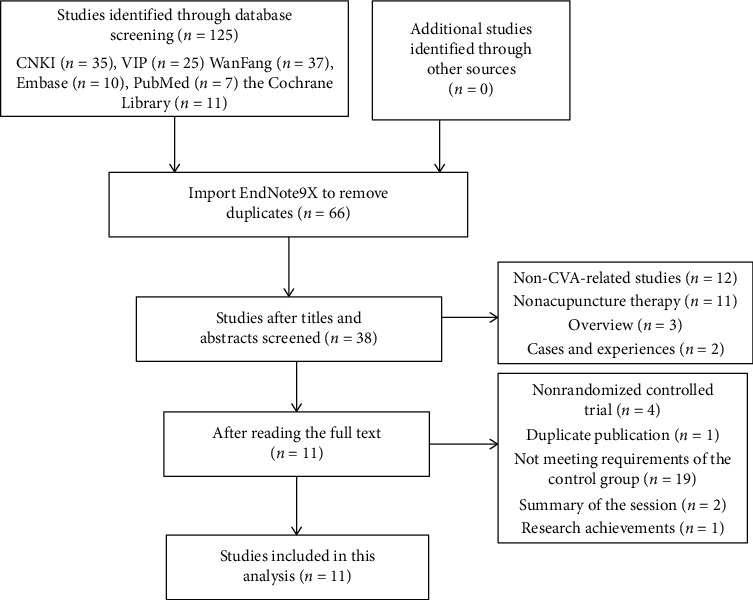
Literature screening flow chart.

A total of 125 related studies were retrieved, including 37 from WanFang, 35 from CNKI, 25 from Weipu, 10 from Embase, 7 from PubMed, and 11 from the Cochrane Library. According to the inclusion and exclusion criteria, 66 studies were selected after repeated retrieval, with 12 non-CVA-related studies, 11 nonacupuncture therapies, 3 reviews, and 2 cases with experience cases and experiences. Of these, 38 were selected after reading the title and abstract. After further reading the full text of the study, there were 4 RCTs, 1 repeated publication of data, 19 failed to meet the requirements of the experimental group and control group, 2 were conference summaries, and 1 was a scientific research result. A total of 11 studies [8–18] that met the standards were finally included in the meta-analysis (Figure 1).

### 3.2. Included in the Survey

The included studies were published since 2012, with a total of 929 CVA patients. They comprised 11 RCT studies from seven Chinese provinces, with patients aged 1 to 82, from the clinic or hospital. Each study baseline was comparable. The acupuncture treatment group had 464 cases, and the control group had 465 cases. Interventions: four [8, 10, 12, 14] studies were designed as acupuncture vs Western medicine, 3 [11, 15, 18] studies were designed as acupuncture and Western medicine vs Western medicine, 3 [13, 16, 17] studies were designed as acupuncture and Chinese medicine vs Traditional Chinese medicine, and 1 [9] study design was for acupuncture plus rehabilitation training vs function rehabilitation training (Table 2).

### 3.3. Risk Bias Assessment and Quality Assessment Included in the Study

**Figure 2:**
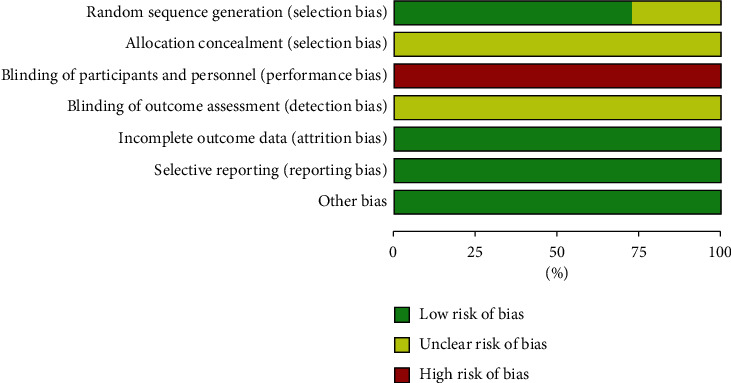
The overall risk bias assessment chart of the included literature.

**Figure 3:**
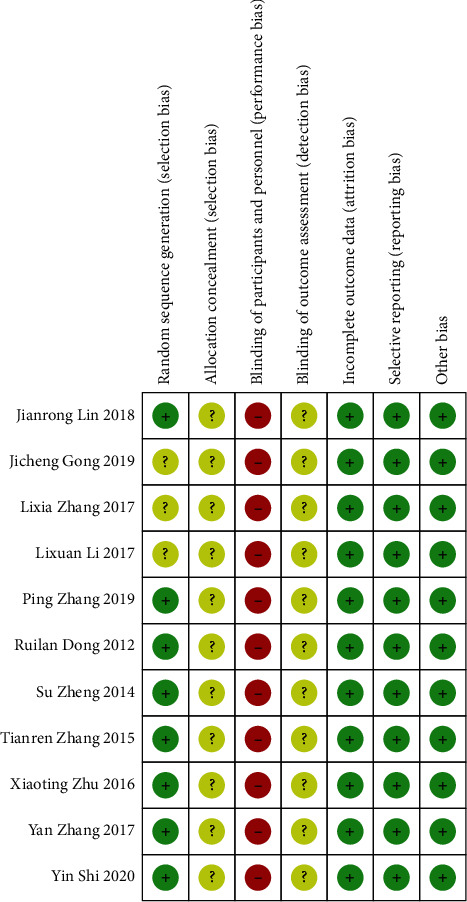
The chart of risk bias assessment of a single item in the included literature.

According to the analysis of the included research literature according to the risk assessment tool, the overall risk of most of the literature was moderate in terms of random sequence generation. All 11 studies were RCTs. The random number table method was adopted in 9 [8, 10, 13–15, 18] studies, 2 [9, 12] studies were randomized, and the remaining studies did not describe the method and referred only to randomness. Concealment aspects of allocation schemes: none of the 11 studies described whether the studies implemented allocation concealment or specific schemes. Blindness: none of the 11 studies described how the researchers or subjects were blinded. In terms of blind evaluation of outcomes, none of the 11 studies mentioned whether the outcome evaluators were blinded or designed. In terms of the completeness of outcome data, 6 [10, 11, 13–15, 18] studies mentioned subjects withdrawal and fall-off and described the reasons. Risk assessment for reporting bias: all 11 studies reported prestated outcomes. In terms of other biases, the baseline levels of the 11 studies were comparable, and there were no other significant biases. In terms of the included study quality evaluation, it was difficult to camouflage and hide acupuncture as a foreign intervention to blind patients and doctors, but the overall quality was medium, with 5 studies of high quality [10, 13–15, 18], 4 studies of medium quality [8, 9, 11, 12] and 2 studies of low quality [16, 17] (Table 3 and Figures 2 and 3 ).

### 3.4. Analysis of the Total Effective Rate of Acupuncture Treatment for CVA

**Figure 4:**
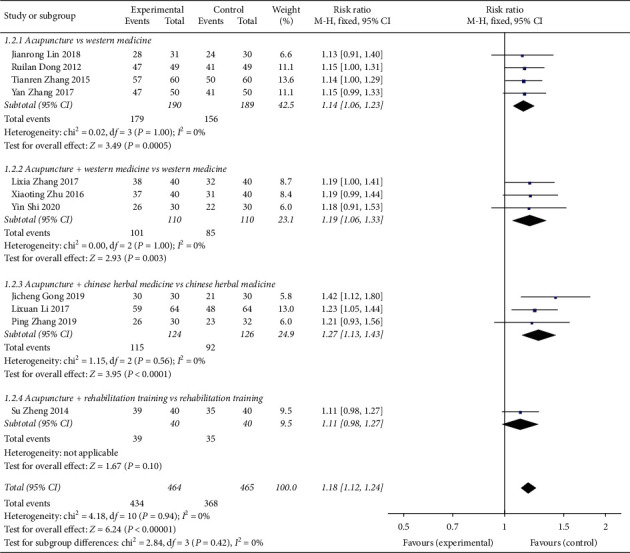
Forest plot for the total effective rate of acupuncture treatment for CVA.

**Figure 5:**
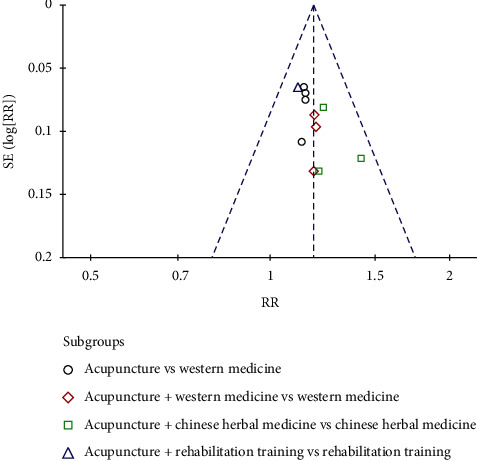
Funnel plot of the total effective rate of CVA treated by acupuncture.

Eleven studies reported total effectiveness (Figure 4). The heterogeneity between studies was *χ*^2^ = 4.18, *P*=0.94, and *I*^2^ = 0%, and the homogeneity between studies was found to be good. The fixed-effects model was used to analyse RR = 1.18, 95% CI (1.12, 1.24), *Z* = 6.24, *P* < 0.00001. The total effective rate of CVA treatment in the supportive acupuncture group was better than that in the nonacupuncture intervention group. The differences in the four acupuncture vs Western medicine subgroups were statistically significant (RR = 1.14, 95% CI (1.06, 1.23)). For acupuncture + Western medicine vs Western medicine, the subgroup analysis in the three studies showed statistically significant differences (RR = 1.19, 95% CI (1.06, 1.33)). The subgroup analysis in the three studies showed statistically significant differences (RR = 1.27, 95% CI (1.13, 1.43)). For acupuncture + rehabilitation training vs rehabilitation training, subgroup analysis in one study showed no statistically significant difference (RR = 1.11, 95% CI (0.98, 1.27)). The funnel plot (Figure 5) is symmetrical on the left and right sides, mainly concentrated on the upper and middle parts, with some risk of publication bias.

### 3.5. Analysis of Recurrence Rate after Withdrawal of CVA by Acupuncture

**Figure 6:**
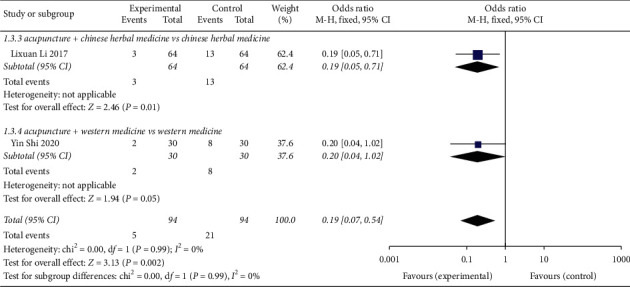
Forest plot of the meta-analysis of CVA recurrence rate in acupuncture treatment.

Two studies [15, 17] reported the recurrence rate of CVA treated by acupuncture (Figure 6), for a total of 188 patients. Good interhomogeneity was found in the included studies (*χ*^2^ = 0.00, *P*=0.99, *I*^2^ = 0%), and good interhomogeneity was found among the studies. The fixed-effects model was used to analyse RR = 0.19, 95% CI (0.07, 0.54), *Z* = 3.13, *P*=0.002. The CVA recurrence rate supported by acupuncture was better than that of the control group. In one subgroup analysis of acupuncture + Chinese herbal medicine vs Chinese herbal medicine, the difference was statistically significant (RR = 0.19, 95% CI (0.05, 0.71)). In one research subgroup, the difference was not statistically significant for acupuncture + Western medicine vs Western medicine (RR = 0.20, 95% CI (0.04, 1.02)).

### 3.6. Integral Analysis of CVA Symptoms in Acupuncture Treatment

#### 3.6.1. Scores of CVA Cough Symptoms Treated by Acupuncture

**Figure 7:**
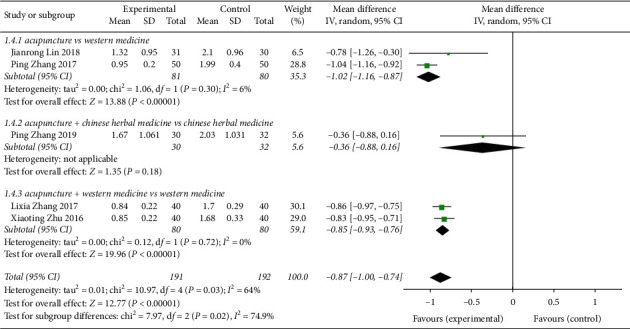
Forest plot of the meta-analysis of CVA cough symptom scores treated by acupuncture.

Five studies [8, 11, 13, 14, 18] reported acupuncture treatment for CVA cough symptom scores (Figure 7). A total of 383 patients were treated with heterogeneity *P*=0.03, *I*^2^ = 64%. A random-effects model was used to analyse the effect size of each study data (MD = -0.87, 95% CI (−1.00, −0.74)), *Z* = 12.77, *P* < 0.00001, indicating that acupuncture was superior to the non-acupuncture intervention control group in relieving CVA cough symptoms. Subgroup analysis indicated that the differences for 2 studies in acupuncture vs Western medicine were statistically significant (MD = -1.02, 95% CI (−1.16, 0.87)); 1 study about acupuncture + Chinese herbal medicine vs Chinese herbal medicine showed significance (MD = -0.36, 95% CI (−0.88, 0.16)). For acupuncture + Western medicine vs Western medicine in 1 study, the difference was statistically significant (MD = −0.87, 95% CI (−0.93, 0.76)).

#### 3.6.2. Scores of CVA Cough Symptoms Treated by Acupuncture

**Figure 8:**
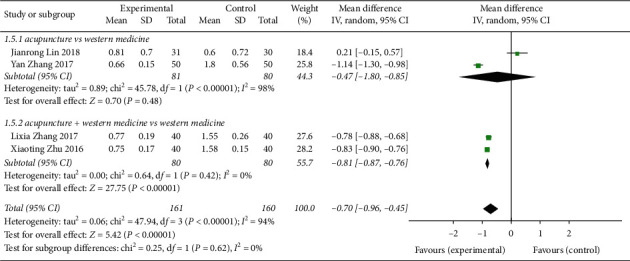
Forest plot of meta-analysis of CVA cough symptom scores with acupuncture treatment.

Four studies [8, 11, 14, 18] reported the cough symptoms of CVA treated by acupuncture, with a total of 321 patients with heterogeneity (*P* < 0.00001, *I*^2^ = 94%). The study data were combined with the random response model (MD = −0.70, 95% CI (−0.96, 0.45)), *Z* = 5.42, *P* < 0.00001, indicating that the cough symptom score of CVA treated by acupuncture was better than that of the control group (Figure 6). Two studies in subgroup analysis for acupuncture vs Western medicine had statistically significant differences (MD = −0.47, 95% CI (−1.80, 0.85)), whereas 2 studies of acupuncture + Western medicine vs Western medicine had statistically significant differences (MD = −0.81, 95% CI (−0.87, 0.76)) (Figure 8).

#### 3.6.3. Score of CVA Diaphragmatic Fullness Symptoms Treated by Acupuncture

**Figure 9:**
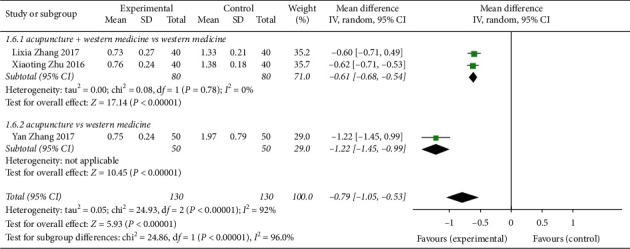
Forest plot of the CVA diaphragmatic fullness symptom score after acupuncture treatment.

Three [8, 11, 18] studies described the indicators of diaphragmatic fullness symptom score (Figure 9), and the differences were heterogeneous (*P* < 0.00001, *I*^2^ = 92%). The data were analysed using a random response model (MD = -0.79, 95% CI (−1.05, −0.53)), *Z* = 5.93, *P* < 0.00001, which indicated that acupuncture treatment of CVA symptoms was superior to the control group in terms of score indicators. The 2 research subgroups had statistically significant differences (MD = −0.61, 95% CI (−0.68, −0.54)). For acupuncture vs Western medicine, 1 research subgroup analysis presented statistical significance (MD = −1.22, 95% CI (−1.45, −0.99)).

### 3.7. Analysis of Lung Function in CVA Treated by Acupuncture

#### 3.7.1. PEF Analysis of the Pulmonary Function Index of CVA Treated by Acupuncture

**Figure 10:**
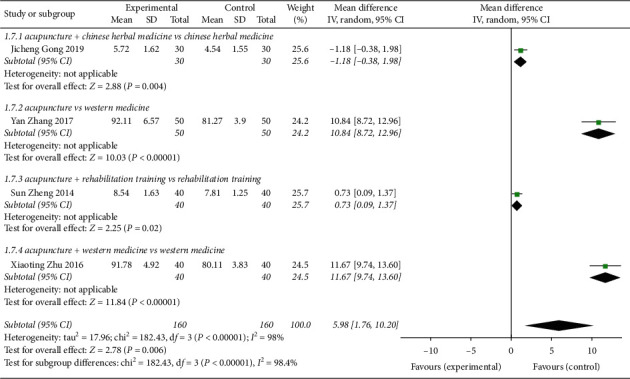
Forest plot of PEF, pulmonary function index of CVA treated by acupuncture.

Four studies [8, 9, 16, 18] had a total of 260 patients with PEF measurements (Figure 10). The heterogeneity test *P* < 0.00001, *I*^2^ = 98%, showed larger heterogeneity among different studies using a random-effects model combined analysis (MD = 5.98, 95% CI (1.76, 10.20), *Z* = 2.78, *P* < 0.00001). For the acupuncture group compared with the control group, the forest plot indicates clear differences between the experimental group and control group for acupuncture treatment of CVA for the lung function index PEF. There was 1 research subgroup of acupuncture + Chinese herbal medicine vs Chinese herbal medicine that had a statistically significant difference (MD = 1.18, 95% CI (0.38, 1.98)). For acupuncture vs Western medicine, there was 1 research subgroup with statistically significant differences (MD = 10.84, 95% CI (8.72, 12.96)). For acupuncture + rehabilitation training vs rehabilitation training, 1 research subgroup had statistically significant differences (MD = 0.73, 95% CI (0.09, 1.37)). For acupuncture + Western medicine vs Western medicine, the difference in 1 research subgroup was statistically significant (MD = 11.67, 95% CI (9.74, 13.60)).

#### 3.7.2. Analysis of Lung Function Index FVC in Acupuncture Treatment of CVA

**Figure 11:**
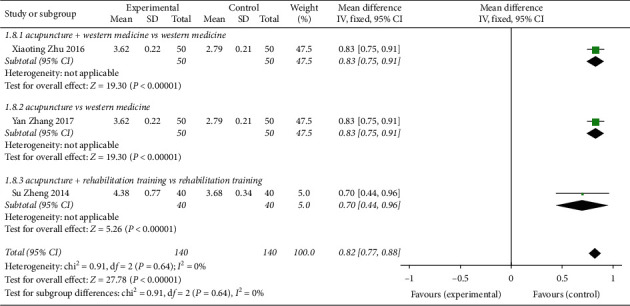
Forest plot of the meta-analysis of FVC and pulmonary function index of CVA treated by acupuncture.

Three [8, 9, 18] studies described the pulmonary function index FVC (Figure 11). The randomized effect model was used to analyse the combined effect amount of the study (MD = 0.82, 95% CI (0.77, 0.88)), indicating that acupuncture treatment was superior to the control group in improving the pulmonary function index FVC, *Z* = 27.78, *P* < 0.00001. For acupuncture + Western medicine vs Western medicine, the difference in 1 research subgroup was statistically significant (MD = 0.83, 95% CI (0.75, 0.91)). One research subgroup for acupuncture vs Western medicine had a statistically significant difference (MD = 0.83, 95% CI (0.75, 0.91)). There was 1 research subgroup with a statistically significant difference for acupuncture + rehabilitation training vs rehabilitation training (MD = 0.70, 95% CI (0.44, 0.96)).

#### 3.7.3. Analysis of the Lung Function Index FEV1 in Acupuncture Treatment of CVA

**Figure 12:**
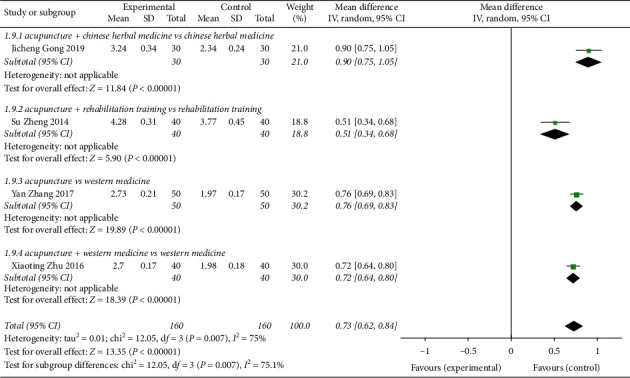
Forest plot of meta-analysis on pulmonary function index FEV1 for CVA treated with acupuncture.

Four studies [8, 9, 16, 18] reported the CVA lung function indicator FEV1 with acupuncture treatment (Figure 12), for a total of 320 patients. The heterogeneity test revealed heterogeneity among studies (*P*=0.007, *I*^2^ = 75%). The random-effects analysis model performed effect size combination analysis on the study data (MD = 0.73, 95% CI (0.62, 0.84), *Z* = 13.35, *P* < 0.00001), indicating that acupuncture can better improve the lung function index FEV1 of CVA compared with the control group. There was 1 research subgroup of acupuncture + Chinese herbal medicine vs Chinese herbal medicine that had statistically significant differences (MD = 0.90, 95% CI (0.75, 1.05)). There was 1 research subgroup with a statistically significant difference for acupuncture + rehabilitation training vs rehabilitation training (MD = 0.51, 95% CI (0.34, 0.68). For acupuncture vs Western medicine, 1 research subgroup analysis difference was statistically significant (MD = 0.76, 95% CI (0.69, 0.83)). For acupuncture + Western medicine vs Western medicine, there was 1 research subgroup with a statistically significant difference (MD = 0.72, 95% CI (0.64, 0.80)).

### 3.8. Biochemical Index Analysis of Acupuncture Treatment for CVA

#### 3.8.1. Acupuncture Treatment of CVA Biochemical Index CRP

**Figure 13:**
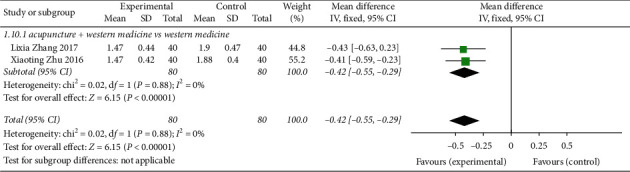
Forest plot of CRP and biochemical index of CVA treated by acupuncture.

Two studies [11, 18] reported CRP, a CVA indicator in acupuncture treatment (Figure 13), with a total of 160 patients. Heterogeneity tests showed good homogeneity among studies (*P*=0.88, *I*^2^ = 0%). The fixed-effects analysis model was used to analyse the two study datasets in combination with dose (MD = −0.42, 95% CI (−0.55, −0.29), *Z* = 6.15, *P* < 0.00001), indicating that acupuncture could better improve the CVA pulmonary function index CRP compared with the control group.

#### 3.8.2. Acupuncture Treatment of CVA Biochemical Index TNF-*α*

**Figure 14:**
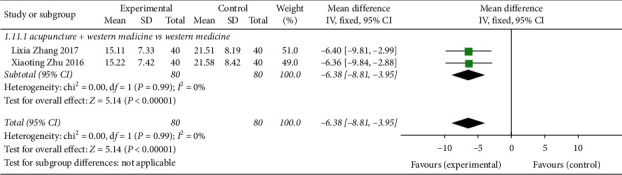
Forest plot of CRP and biochemical index of CVA treated by acupuncture.

Two studies [11, 18] reported acupuncture therapy for the CVA indicator TNF-*α* (Figure 14), involving a total of 160 patients. The heterogeneity test found that the heterogeneity between studies was good (*P*=0.99, *I*^2^ = 0%). The fixed-effects analysis model was applied to carry out combined effect analysis on the data of the two studies (MD = −6.38, 95% CI (−8.81, 3.95), *Z* = 5.14, *P* < 0.00001), indicating that acupuncture could better improve the CVA lung function index FEV1 compared with the control group.

#### 3.8.3. Acupuncture Treatment of CVA Biochemical Index IgE

**Figure 15:**
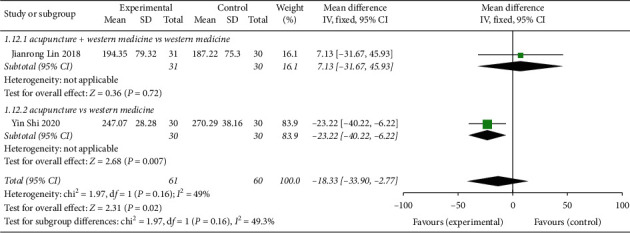
Forest plot of CVA biochemical indicator IgE with acupuncture treatment.

Two studies [14, 15] reported acupuncture treatment of the CVA indicator IgE (Figure 15), with a total of 121 patients. The heterogeneity test found good homogeneity among the studies (*P*=0.16, *I*^2^ = 49%). The fixed-effects analysis model was used to combine the effects of two studies (MD = −18.33, 95% CI (−33.90, −2.77), *Z* = 2.31, *P*=0.02). Compared with the control group, acupuncture treatment significantly improved CVA standard IgE. There was 1 research subgroup with no significant difference for acupuncture + Western medicine vs Western medicine (MD = 7.13, 95% CI (−31.67, 45.93)). For acupuncture vs Western medicine, 1 subgroup analysis had a statistically significant difference (MD = −23.22, 95% CI (−40.22, −6.22)).

### 3.9. Adverse Reactions

Among the 14 studies, 6 [10, 13–15] mentioned no adverse reactions between the experimental group and the control group, and 3 mentioned adverse reactions: Yan [8] acupuncture group: gastrointestinal reactions (2 cases), control group: liver function impairment (4 cases), renal function impairment (4 cases), routine blood abnormalities (2 cases), gastrointestinal reactions (6 cases), and hypersensitivity reactions (2 cases). Gong [16] reported two cases of nausea in the TCM group, two cases of nausea in the acupuncture + TCM group, and two cases of local skin redness. Li [17] showed that the acupuncture plus Chinese medicine group (1 case) vs the Chinese medicine group (11 cases) showed fewer adverse reactions and higher safety in the treatment of CVA with acupuncture.

## 4. Discussion

At present, modern studies have not fully defined the pathogenesis and pathological process of cough-variant asthma, and the mainstream view is that CVA has histopathological changes similar to those of conventional asthma: eosinophilic airway inflammation, bronchohyperreactivity (BHR) [19] and airway remodelling. A variety of inflammatory cells, cytokines, and inflammatory mediators participate in and interact with each other to contribute to the nonspecific chronic inflammatory response of this disease, which indirectly shows characteristic airway hyperresponsiveness and cough receptor hypersensitivity, cell infiltration, and gene expression of inflammatory cytokines. [20] Acupuncture has important potential and advantages in treating cough-variant asthma. Numerous clinical studies and animal experiments have found that acupuncture can improve pulmonary ventilation function and anti-inflammatory activity, enhance immunity of the body, regulate the neuroendocrine network and other aspects, and treat asthma through multiple channels, levels, links, and two-way regulation. [21] Yang Yongqing's team at Shanghai University of Traditional Chinese Medicine also made important progress in basic research on the therapeutic effects of acupuncture on asthma to explore anti-asthma target discovery, which further demonstrated the effectiveness of acupuncture therapy [22, 23].

In this study, a total of 929 patients (464 cases in the treatment group and 465 cases in the control group) were included in 11 RCT studies of acupuncture treatment for CVA. Meta-analysis results based on published evidence showed that total effective rate, recurrence rate, and symptom integral (cough, sputum, XiongGe full tightness), pulmonary function (FVC, FEV1, and PEF), and biochemical indicators (CRP, TNF alpha, and IgE) were superior with acupuncture treatment of CVA compared to control groups. The results showed that giving priority to simple acupuncture or auxiliary treatment of CVA clinical curative effect is better. Advantages include reducing the recurrence rate, improving symptoms, and regulating immune inflammation index. The quality of the studies was low, but there was research into the negative reports and risk of bias. Safety analysis: 3 of the 11 studies mentioned adverse reactions of acupuncture, mainly manifesting as minor reactions such as gastrointestinal reactions and local skin redness. Compared with the control group, there were fewer adverse reactions, and the safety was higher.

Nevertheless, there are some limitations of this research. First, although the acupuncture treatment of CVA is common, the clinical randomized controlled study sample size was small, and the design was not rigorous. The curative effect evaluation method has problems, such as measurement standards that are not unified, risk bias, and clinical heterogeneity; 10 studies included patients who were from China and single-centre studies. As a result, it is difficult to accurately describe the efficacy of acupuncture therapy in the treatment of this disease. It also indicates that it is urgent to adopt high-quality clinical research methods in CVA acupuncture research, adopt correct randomized methods, allocate hidden and double-blind methods, and adopt recognized and unified efficacy evaluation standards to carry out large multicentre sample studies. Researchers should follow CONSORT clinical trial reporting standards to improve the quality of RCT reporting. In terms of the selection of acupuncture intervention measures and study outcome indicators, standardized reports should be made according to the guidelines and STRICTA clinical intervention report standard. Second, the quality of the included literature was generally not high. None of the 11 studies described or reported the implementation of allocation and concealment. It is suggested that attention should be paid to these factors in future studies. In terms of the implementation of blinding methods, acupuncture therapy is special, and it is difficult to achieve double blindness. However, blind methods can be applied in the evaluation of outcome indicators to reduce bias. Third, due to the particularity of acupuncture therapy, this study only focuses on acupuncture stimulation methods and does not analyse the differences in acupuncture acupoint selection, manipulation, penetration depth, and course of treatment to deeply discuss the curative effect of acupuncture. Fourth, the literature retrieval language was only selected for Chinese and English literature, mainly from 6 database resources.

## 5. Conclusion

Acupuncture therapy in cough-variant asthma is efficient, and the recurrence rate was superior to the acupuncture group. For alleviating symptoms of cough, sputum, and XiongGe nausea, and improving the lung function index and immune inflammation index, acupuncture has positive significance. For acupuncture physicians in clinical treatment, the evidence supports that acupuncture can be considered in the treatment of cough-variant asthma of auxiliary means. Future efforts still need more high-quality, multicentre, large sample, randomized, double-blind, placebo-controlled trials to improve the quality of the methodology and reporting.

## Figures and Tables

**Table 1:** Embase: session results.

No.	Query results	Results	Date

#5.	#3 AND #4	10	23 Nov 2020

#4.	#1 OR #2	49, 173	23 Nov 2020

#3.	“cough-variant asthma”:ab, ti OR “cough type	6, 908	23 Nov 2020
	asthma”:ab, ti OR cva: ab, ti		

#2.	“acupuncture therapy”/exp OR	49, 173	23 Nov 2020
	electroacupuncture: ab, ti OR “electroacupuncture		
	therapy”: ab, ti OR “manual acupuncture”: ab, ti OR		
	“dry needle”: ab, ti OR acupoint: ab, ti		

#1.	“acupuncture”/exp	48, 448	23 Nov 2020

**Table 2:** Basic information of included studies.

Author	Interventions	Control group	The number of cases/case		Gender (male/female)	Age		Period of treatment (d)	Diagnostic criteria and efficacy criteria	Outcome indicators		Follow-up time	Region	
			Experimental group	Control group																
Zhang 2017 [8]	⑩	① + ②	50	50	63/37	18–60		56	(1), (2)	Symptom score (cough score, sputum score, diaphragmatic fullness score), lung function (PEF, FVC, FEV1), total effective rate, adverse reactions		Not mentioned	China's Hebei province	

Zheng 2014 [9]	⑩ + ③	③	40	40	57/62	15–67		10 d	(1), (3)	Total response rate, lung function (FVC, FEV1, PEF), general adaptive quality of life questionnaire dimensions and total scores, specific quality of life questionnaire scores in all areas, and LCQ total scores		Not mentioned	China's Hubei province	

Zhang 2015 [10]	⑩	① + ④	60	60	67/53	6–52		90	(4)	Total effective rate		Not mentioned	China's Liaoning province	

Zhang 2017 [11]	⑩ + ① + ⑤ + ⑥	① + ⑤ + ⑥	40	40	48/32	18–59		28	(1), (2), (3)	CRP, IL-6, TNF-, total response rate, symptom score (cough, sputum, diaphragmatic fullness)		Not mentioned	China's Hebei province	

Dong 2012 [12]	⑩	① + ②	49	49	34/64	19–72		14	(4)	Total effective rate, time for improvement of clinical symptoms and signs (dry cough, expectoration, chest tightness, wheezing)		Not mentioned	China Ningxia	

Zhang 2019 [13]	⑩ + ⑨	⑨	30	32	32/30	1–14		14	(2), (7), (8), (9), (10), (11)	Cough symptom score, (self-modified) TCM syndrome score, effective rate of cough symptom score, effective rate of TCM syndrome score, 3-month recurrence rate, 3-month follow-up score of digestive tract symptoms		3 months	China's Fujian province	

Lin 2018 [14]	⑩	④ + ⑤	31	30	24/37	18–70	14		(2), (10), (12)		Total effective rate, score of cough symptoms, score of comparison of symptoms and signs, total score of symptoms, IgE, safety index	Not mentioned	China's Fujian province

Shi 2020 [15]	⑩ + ①	①	30	30	31/41	3–12		28	(2), (8), (9)	Total effective rate, IgE, TCM symptom score, 6-month recurrence rate, safety index		6 months	China's Fujian province	

Gong 2019 [16]	⑩ + ⑨	⑨	30	30	39/21	62–82		30	Describe	Total effective rate, IgA, IgG, lung function (PEF, FEV1), cough disappearance time, length of hospital stay, adverse reactions		Not mentioned	China's Sichuan province	

Li 2017 [17]	⑩ + ⑨	⑨	64	64	67/61	59–82		21	Describe	Total effective rate, duration of asthma, time of disappearance of cough, time of disappearance of pulmonary wheezing, adverse reactions, recurrence rate, adverse reactions		Not mentioned	China's Guangdong province	

Zhu 2016 [18]	⑩ + ① + ⑤ + ⑥	① + ⑤ + ⑥	40	40	48/32	18–57		28	(1), (2), (3)	CRP, IL-8, TNF-, total response rate, symptom score (cough, sputum, diaphragmatic fullness), pulmonary function (FVC, FEV1, PEF)		Not mentioned	China's Hebei province	

Note: ① montelukast sodium; ② cloth DE resistance; ③ pulmonary function rehabilitation training; ④ forticasone propionate; ⑤ salmeteroticasone; ⑥ terbutaline; ⑦ albuterol; ⑧ aminophylline; ⑨ traditional Chinese medicine medicinal broth; ⑩ acupuncture. Diagnostic and therapeutic criteria: ⑴ 2009 Guidelines for Diagnosis and Treatment of Cough; ⑵ Guiding Principles for Clinical Research of New Chinese Medicines; (3) Diagnostic and Curative Effect Criteria of TCM Diseases and Syndromes; ⑷ Guidelines for the Prevention and Treatment of Bronchial Asthma; ⑸ Guide to Diagnosis and Treatment of Chronic Cough in Children (2013); ⑹ “TCM Clinical Diagnosis and Treatment Terminology Syndrome Part”; ⑺ Paediatrics of Traditional Chinese Medicine; ⑻ Guidelines for the Diagnosis and Treatment of Bronchial Asthma in Children; ⑼ Clinical Diagnosis and Treatment Guidelines for Chinese Medicine Paediatrics Children's Cough-Variant Asthma (developed); ⑽ 2015 Diagnostic and Treatment Guidelines for Cough; ⑾ Clinical Research Points of Traditional Chinese Medicine for Paediatric Diseases; ⑿ Practical Traditional Chinese Medicine Internal Medicine.

**Table 3:** Included research risk assessment and quality assessment.

Author	Random sequence generation	Allocation scheme hiding	Blind method	Blind method evaluation of the outcome	Result data integrity	Selective reporting of research findings	Other sources of bias	The Jadad score
Zhang 2017 [8]	Random number table, low	Dimness	Dimness	Dimness	Dimness	No, low	Low	2
Zheng 2014 [9]	The order of visits was random and low	Dimness	Dimness	Dimness	Dimness	No, low	Low	2
Zhang 2015 [10]	Random number table, low	Dimness	Dimness	Dimness	Complete, low	No, low	Low	3
Zhang 2017 [11]	Yes, dimness	Dimness	Dimness	Dimness	Complete, low	No, low	Low	2
Dong 2012 [12]	The order of visits was random and low	Dimness	Dimness	Dimness	Dimness	No, low	Low	2
Zhang 2019 [13]	Random number table, low	Dimness	Dimness	Dimness	Complete, low	No, low	Low	3
Lin 2018 [14]	Random number table, low	Dimness	Dimness	Dimness	Complete, low	No, low	Low	3
Shi 2020 [15]	Random number table, low	Dimness	Dimness	Dimness	Complete, low	No, low	Low	3
Gong 2019 [16]	Yes, dimness	Dimness	Dimness	Dimness	Dimness	No, low	Low	1
Li 2017 [17]	Yes, dimness	Dimness	Dimness	Dimness	Dimness	No, low	Low	1
Zhu 2016 [18]	Random number table, low	Dimness	Dimness	Dimness	Complete, low	No, low	Low	3

## Data Availability

The data used to support the findings of this study are included within the article.
